# Editorial: Stroke in minority groups and populations

**DOI:** 10.3389/fstro.2023.1315298

**Published:** 2023-11-28

**Authors:** Narayanaswamy Venketasubramanian, Tatjana Rundek, Robert N. Gan

**Affiliations:** ^1^Raffles Neuroscience Centre, Raffles Hospital, Singapore, Singapore; ^2^Department of Neurology and Evelyn F. McKnight Brain Institute, Miller School of Medicine, University of Miami, Miami, FL, United States; ^3^Philippine Neurological Association One Database, Quezon City, Philippines

**Keywords:** stroke, minorities, disparities, pediatric stroke in COVID-19, young stroke, readmission after stroke

To reduce the overall burden of stroke, effective interventions must be implemented across all groups and populations that are at risk of or suffer a stroke (Feigin et al., 2020). While many research and clinical trials are conducted on stroke patients in general, certain groups or populations are under-represented and less studied but may need a more tailored approach (Sacco, 2020). Strokes happen in members of minority groups that include, but are not limited to, children, people over 80 years old, women, pregnant women, transgender people, racial minorities, native/Indigenous people, people in lower economic brackets, migrant workers, prisoners, unemployed people, athletes, and residents of communities under threat (Trimble and Morgenstern, 2008). They may be socially marginalized, at a particularly higher risk of stroke, have different and unusual underlying causes requiring different treatments, or would experience a bigger socio-economic impact if they suffered a stroke.

Among the trail-blazers in the study of stroke disparities and effects on minorities was Dr. Ralph L Sacco, who was Olemberg Family Chair in Neurological Disorders, Miller Professor of Neurology, Public Health Sciences, Human Genetics, and Neurosurgery at the Leonard M. Miller School of Medicine at the University of Miami and Chief of the Neurology Service at Jackson Memorial Hospital, prior to his untimely demise on 17 January 2023. He was a tutor, colleague, mentor, and dear friend to the authors of this Editorial—it is to him that this Editorial is dedicated. His seminal project and life's work was the NOrthern MAnhattan Study (NOMAS). He evaluated race and ethnic differences in stroke between three different race-ethnic groups living in an urban multi-ethnic community (Sacco, 2020). NOMAS demonstrated differences in stroke risk factors, incidence, subtypes, and outcomes, possibly explained by disparities in ideal cardiovascular health, highlighting the need for tailored risk factor modification in minorities to achieve this ideal (Sacco, 2011).

Disparities in disease burden exist, which may be defined by race/ethnicity, sex, age, geography, and socioeconomic status (Elkind et al., 2020). The Maori, the Indigenous people of Aotearoa (New Zealand), develop stroke 10 to 15 years earlier, at a higher rate, than New Zealanders of European descent. Ranta et al. explored how these differences may be related to differential exposure to stroke risk factors, access to early diagnosis and treatment, and unequal treatment in stroke unit care. There may be fiscal constraints and socio-economic challenges. The root causes for these ethnic inequities included unconscious bias, racism, culturally unsafe treatment environments, and culturally incongruent treatment approaches. Health system reforms with five key objectives have been proposed: equity, tino rangatiratanga (self-determination), options, partnership, and active protection of those rights. The emphasis would be on wellbeing and prevention. Maori leadership, research including partnering with non-Maori, and incorporation of traditional Maori knowledge, practices, and preferences are needed.

“Falling” stroke incidence rates may not be mirrored in disadvantaged, minority populations. This may be due to inappropriate methods used for measuring incidence in these populations. Balabanski et al. compared three studies of stroke incidence in Aboriginal Australians that used different methods for case ascertainment. The “gold standard” population-based method captured both out-of-hospital and in-hospital stroke events but had few Aboriginal patients. A retrospective hospital-based cohort design provided a larger sample size that allowed stroke subtyping but suffered selection bias, as it was limited to hospitalized cases. Whole-of-population linked hospital and mortality data had a large sample size and allowed for subgroup analysis, but it lacked clinical adjudication and had large proportions of “undetermined stroke.” Still, despite diagnostic imprecision, the authors recommend the use of whole-of-population data linkage including non-hospitalized stroke deaths when measuring stroke incidence in Indigenous, minority populations.

The recent COVID-19 pandemic greatly stressed healthcare systems globally. Layug et al. performed a systematic review of the mechanisms underlying pediatric ischemic stroke (IS) in COVID-19 pandemic. Of 74 patients included, 51.5% were female, and mean age was 9.2 ± 5.6 years. Stroke incidences were largely arterial strokes (82.4%), with fewer cerebral venous sinus thrombosis (12.2%), or a combination of arterial and venous strokes (5.4%). Mechanisms included thrombophilia (47.3%) and vasculopathies (27%), with some due to cardioembolism (6.8%). Only 27% had illnesses that predisposed them to stroke; 18.9% had Multi-system Inflammatory Syndrome in Children. While 22.4% recovered fully, 60.3% had residual deficits, and 17.2% died. This contrasts with a systematic review of 168 adults, 58.9% male, median age 53 years (range 25–89 years), where 85.7% had IS, 76.8% had at least one cerebrovascular risk factor, and 12% had cardioembolic stroke (Frisullo et al., 2021). In another systematic review of 54 adults with IS, 62.3% male, mean age 63.4 ± 3.1 years, 13.0% had cardioembolic stroke, and mortality was 38% (Tan et al., 2020).

The impact of stroke is higher in disabled younger survivors than older adults, as they are affected during their most productive years. Ignacio et al. studied 114 young adults in the Philippines, mean age 39.4 years, at a mean of 4 months after ischemic (58.8%) or hemorrhagic (41.2%) stroke, using the European Quality of Life Five Dimension Five Level Scale (EQ-5D-5L). They found that those with both anxiety and depression had the lowest ratings on the Heath-Related Quality of Life (QoL) scales, with an EQ-Visual Analog Scale of 60 vs. 90 (*p* = 0.01) and an EQ-5D summary index of 0.64 vs. 0.89 (*p* < 0.01) when compared to those without both conditions. Anxiety and depression were significantly correlated with poor QoL on all dimensions of the EQ-5D-5L, and Barthel Index with problems in mobility (OR 0.17, 95%CI 0.03–1.02) and self-care (OR 0.08, 95%CI 0.01–0.55). A scoping review of nine papers on young stroke and age-matched controls from the general population showed that QoL was correlated with the modified Rankin scale, Barthel index, and post-stroke fatigue and depression (Gurková et al., 2023).

Hospital readmission after stroke may be an indicator of poor transition to post-stroke care. A meta-analysis and systematic review of 17 retrospective observational studies (*n* = 1,829,964) revealed a 10.66% ± 6.87% (range 1.41–27.64%) 30-day readmission rate of ischemic stroke (Deng et al., 2021). Risk factors were history of stroke, diabetes mellitus, hypertension, atrial fibrillation, heart failure, and age. Gardener et al. used data from the multicenter Florida Stroke Registry, which included 45,877 patients discharged home or to rehabilitation centers with an ischemic stroke or intracerebral hemorrhage between 2017 and 2019. Mean age was 68 ± 14 years, 46% were women, 64% non-Hispanic White, 21% non-Hispanic Black, and 15% Hispanic. Hospital readmission within 30 days was 12%, with 6% vascular-related and 3% from recurrent stroke. Readmission was independently associated with Medicare or Medicaid insurance, large artery atherosclerosis as the stroke mechanism, increased stroke severity, diabetes mellitus, atrial fibrillation, peripheral vascular disease, coronary artery disease, prior stroke, chronic renal insufficiency, and depression. Interestingly, decreased risk of all-cause readmission was seen with ambulation, treated dyslipidemia, tPA treatment, discharge mRS 0–2, and treatment at a comprehensive stroke center, indicating that some of these readmissions were preventable.

The goal of this Research Topic is to provide a platform to highlight recent research on the specificities, profiles, clinical features, care, treatment and interventions, and outcomes of stroke patients belonging to these groups or populations, many of which continue to be underrepresented in studies. We note the lack of research and general interest in specific groups in stroke research—much more needs to be done, and adequate funding for research in minority and minorized populations is needed (Sacco et al., 2015). Worldwide, we need access to stroke treatments and rehabilitation, better control of stroke risk factors, and better understanding of social determinants of health at individual and systemic levels. Still, we hope the data presented here, though it may only represent the tip of the iceberg, should help improve our understanding of how these factors impact the overall burden of stroke and what can be done to improve outcomes and brain health for all.

## Author contributions

NV: Conceptualization, Writing—original draft. TR: Conceptualization, Supervision, Writing—review & editing. RG: Conceptualization, Supervision, Writing—review & editing.
